# A patient with Creutzfeldt-Jakob disease presenting with amyotrophy: a case report

**DOI:** 10.1186/1752-1947-7-218

**Published:** 2013-08-23

**Authors:** Peter K Panegyres, Elizabeth Armari, Richard Shelly

**Affiliations:** 1 Neurodegenerative Disorders Research Pty Ltd, 185 York Street, Subiaco, Perth 6008, Western Australia; 2 Neurology Unit, Department of General Medicine, Joondalup Health Campus, Shenton Avenue, Joondalup 6027, Western Australia; 3 Department of General Medicine, Joondalup Health Campus, Shenton Avenue, Joondalup 6027, Western Australia

**Keywords:** Creutzfeldt-Jakob disease, Amyotrophy, Anterior horn cells

## Abstract

**Introduction:**

Creutzfeldt-Jakob disease (CJD) is an ultimately fatal, neurodegenerative disease caused by misfolded prion protein aggregation and accumulation. The development of amyotrophic features has been described in CJD, though rarely as an early or prominent feature. Consequently, the significance of amyotrophy in prion disease etiology remains unclear.

**Case presentation:**

Our patient, a healthy 70-year-old French/Algerian man, presented to our hospital following a work-related fall and was admitted with lower limb skeletal muscle atrophy and fasciculations; the fasciculations progressed to involve the trunk, upper limbs and face. Within days, he developed evidence of a progressive ascending neurological syndrome and subsequent brain involvement with supranuclear palsy of upgaze, catalepsy and death 36 days after symptom onset. Amyotrophy remained the principle feature of his disease. Dementia started to develop within 10 days of the onset of his amyotrophy. Prion disease was confirmed at postmortem.

**Conclusions:**

Our observations suggest an unusual form of prion disease with prominent early involvement of anterior horn cells, ascending prion propagation in the central nervous system and a grave prognosis.

## Introduction

Creutzfeldt-Jakob disease (CJD) is a fatal neurodegenerative disease characterized by rapidly progressive dementia caused by prion protein propagation [1,2]. Amyotrophy is characteristic of amyotrophic lateral sclerosis (ALS), although its presence throughout the clinical course of CJD has been described [3]. However, the significance of lower motor neuron involvement in prion disease etiology is disputed [4,5], and the suggestion of an amyotrophic variant of the disease has been discredited [3,4]. Amyotrophy in CJD has been viewed as a terminal phenomenon in emancipated patients [4]; though Worrall *et al*. [3] highlight the occasional predominance of amyotrophic features throughout the disease course. Patients with the suspicion of a prion disease and amyotrophy are important to study as they potentially expand the clinical spectrum of the prionopathies.

This article charts the clinical course of marked early amyotrophic features from symptomatic onset until our patient’s death. Fasciculations and muscle atrophy remained prominent, with clinical evidence to suggest step-wise, ascending prion propagation from our patient’s lower limbs, trunk, upper limbs and finally brain. A diagnosis of sporadic CJD was confirmed at postmortem.

## Case presentation

A 70-year-old French/Algerian man presented to our hospital following a work-related fall. He had full functional capacity in all acts of daily living including driving, lived independently and was working until the day of his presentation. An initial physical examination revealed the presence of fasciculations in all muscles of the lower limbs, warranting further investigation and admission. The patient had stable non-insulin-dependent diabetes mellitus.

An initial neurological assessment revealed normal cognition, speech and swallowing. An examination of his central and peripheral nervous systems revealed no diagnostic abnormalities, other than the fasciculations in the lower limbs with mild asymmetrical leg weakness (Table 1, Day 1). His mini mental state examination result of 25 and clock drawing test measurement of four out of five were considered within normal limits for a French/Algerian man of age 70 where English was not his first language. Furthermore, his Addenbrooke’s cognitive assessment and total functional capacity were normal. There were no upper motor neuron signs and the patient could walk without assistance. An initial magnetic resonance imaging (MRI) scan did not show diagnostic abnormalities in the brain or cervical spine. Nerve conduction studies were normal and an electromyography (EMG) test on day 9 showed widespread active and chronic partial denervation in all skeletal muscle sampled: fibrillation and fasciculation potentials were present in all examined muscles and in all four limbs.

**Table 1:** Cognition and neuromuscular status – amyotrophy in prion disease

		**Hospital day**				
		**Day 1**	**Day 7**	**Day 10**	**Day 16**	**Day 30**
**Cognition:**						
Mini mental state examination		25	-	23	9	0
Clock drawing test		4	-	3	0	0
Total functional capacity		12	-	2	1	0
Addenbrooke’s cognitive assessment (revised)		83	-	72	38	0
• Attention and orientation		16/18	-	13/18	4/18	0
• Memory		22/26	-	20/26	13/26	0
• Fluency		10/14	-	8/14	8/14	0
• Language		22/26	-	20/26	13/26	0
Visuospatial		13/16	-	11/16	0/16	0
**Neuromuscular:**						
Neck	F	5*	5	4	3	2
	E	5	5	4	3	2
Hip	F	4^(L)^, 5^(R)^	3^(L)^, 3^(R)^	3^(L)^, 3^(R)^	3^(L)^, 3^(R)^	2^(L)^, 2^(R)^
	E	4, 5	3, 3	3, 3	3, 3	2, 2
Hip	ABD	4, 5	3, 4	3, 3	3, 3	2, 2
	ADD	4, 5	3, 4	3, 3	3, 3	2, 2
Knee	F	4, 5	3, 3	3, 3	3, 3	2, 2
	E	4, 5	3, 3	3, 3	3, 3	2, 2
Ankle	DF	5, 5	3, 3	3, 3	3, 3	2, 2
	PF	5, 5	4, 4	3, 3	3, 3	2, 2
	INV	5, 5	3, 3	2, 2	2, 2	2, 2
	EV	5, 5	3, 3	2, 2	2, 2	2, 2
Shoulder	ABD	5, 5	5, 5	4, 4	3, 3	2, 2
	F	5, 5	5, 5	4, 4	3, 3	2, 2
	E	5, 5	5, 5	4, 4	3, 3	2, 2
Elbow	F	5, 5	5, 5	3, 3	3, 3	2, 2
	E	5, 5	5, 5	3, 3	3, 3	2, 2
Wrist	F	5, 5	5, 5	3, 3	3, 3	2, 2
	E	5, 5	5, 5	3, 3	3, 3	2, 2
Finger	F	5, 5	5, 5	3, 3	3, 3	2, 2
	E	5, 5	5, 5	3, 3	3, 3	2, 2

*Medical Research Council Scale 0 to 5. *F* Flexion, *E* Extension, *ABD* Abduction, *ADD* Adduction, *PF* Plantar flexion, *INV* Inversion, *EV* Eversion, *DF* Dorsiflexion, *L* Left, *R* Right.

A week following presentation, our patient experienced increased difficulty walking with worsening leg weakness (Table 1, Day 7). The fasciculations had ascended and were now clinically evident in the lumbar and thoracic paraspinal muscles and upper limbs.

Electroencephalography (EEG) revealed nonspecific slow wave changes without epileptiform activity. Our patient’s symptomatic progression continued and by day 10 he developed worsening lower and upper limb weakness with some decrease in his cognitive status (Table 1). On day 16 he reported short-term memory loss and had a supranuclear palsy of upgaze with deteriorating limb weakness. The fasciculations had now ascended to his neck and facial muscles. His tongue, however, remained normal without fasciculations. There was new evidence of a marked cognitive deterioration. Our patient’s limb strength continued to deteriorate and he became progressively bed-bound and confused by day 30.

A second EEG on day 16 revealed increases in slow wave activity suggestive of encephalopathy. Six days later, the EEG demonstrated isolated epileptogenic activity in the left temporal and parietal regions with diffuse periodic discharges. Our patient developed catalepsy as he continued to deteriorate. The 14-3-3 protein was detected in his cerebrospinal fluid, which was otherwise normal and without malignant cells (day 20). A repeat MRI scan on day 22 revealed multiple areas of diffusion-weighted imaging positivity in the basal ganglia, right cingulate gyrus and the anterior border of the right caudate head suggestive of prion disease. He did not have mutations in the prion gene (PRPN) on gene testing and was Met/Met at codon 129. Our patient continued to deteriorate and died 36 days after symptom onset.

A postmortem examination revealed patchy neuronal loss, mild reactive astrogliosis and numerous diffuse neuritic plaques within the brain, with several leptomeningeal and cortical blood vessels positive for beta-amyloid: all compatible with our patient’s age, and without neurofibrillary tangles. Spongiform change was widespread in the neocortex. Immunohistological staining for the 12F10 prion antibody revealed patchy synaptic-like positivity in the cerebral cortex, thalamus, cerebellar cortex and brainstem motor neurons. The brain and spinal cord were macroscopically normal. Our mortuary does not permit spinal cord sampling for prion disease and our neuropathology laboratory does not perform prion protein isotyping. Such observations strongly suggested an unusual form of prion disease with prominent early involvement of anterior horn cells and ascending spinal propagation.

## Discussion

**Figure 1:**
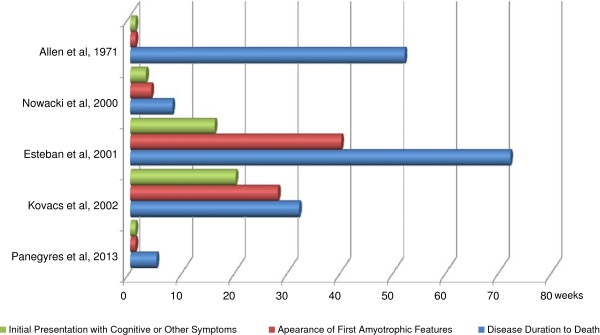
The course of amyotrophy in Creutzfeldt-Jakob disease.

This case report provides an insight into the presentation and manifestations of amyotrophic features in CJD from symptom onset to death. The presence of amyotrophy in CJD has been previously documented [4-7], although mostly as a terminal consequence of the disease [4]. To the best of our knowledge, our patient is the first to have experienced amyotrophic features so early in the disease course and to have symptoms suggestive of ascending prion propagation from lower limbs, trunk musculature, upper limbs and brain (Figure 1). Disease duration is typically six months [3], making the rapidity of our patient’s deterioration and death striking.

Amyotrophy has been described in both familial and sporadic CJD cases [3,5]. However, quantifying the prevalence of lower motor neuron dysfunction in CJD is problematic in the absence of a rigorous definition [3]. Moreover, the diversity of clinical amyotrophic manifestations reported in CJD has raised doubts over the significance of concurrent upper and lower motor neuron involvement in prion disease.

Worrall *et al*. [3] report 13 patients with sporadic CJD who demonstrate clinically relevant lower motor neuron disease with associated electomyographic denervation [3,4], concluding that amyotrophy is occasionally a significant feature of CJD (readers are referred to that work for detail of those patients). However, the clinical progression and neuropathological findings of only three of these patients convincingly portray the emergence of amyotrophic features with an undoubted CJD diagnosis (Table 2) [4,7,8]. Since Worrall *et al.*’s findings [3], reports of amyotrophy in CJD have been scant, with the emergence of lower motor neuron features vague upon a questionable diagnosis. Nevertheless, amyotrophy has been reported in six patients with confirmed CJD [4,5,7,8] (Table 2), although only three provide detailed information of their patient’s clinical course [4,7,8].

**Table 2:** Amyotrophy in Creutzfeldt-Jakob disease – reported patients

**Reference**	**Gender**	**Age at onset**	**Disease duration (months)**	**Initial symptoms**	**Duration of symptoms before presentation**	**Presence of amyotrophy**	**Neuropathology**
Allen *et al*., 1971 [9]	M	56	13	◦ Asymmetrical weakness and wasting of the upper limbs	At onset	First sign at clinical presentation	◦ Frontal cortex neurodegeneration, marked gliosis
				◦ Generalized fasciculations			◦ Spongiform change of temporal lobes
				◦ Memory loss			◦ Loss of anterior horn cells with accompanied gliosis
				◦ Confabulation and constructional ataxia			
Traub *et al*., 1977 [10]	F	35	12	◦ Memory loss	ND	10 months after clinical presentation	◦ Severe spongiform change, astrocytosis in the putamen, caudate nucleus and amygdala
				◦ Diplopia			◦ Degeneration of lateral corticospinal tracts
							◦ Neuronal loss of anterior horn cells
Traub *et al*., 1977 [10]	M	61	9	◦ Memory loss	ND	6 months after clinical presentation	◦ Neurodegeneration of cerebral cortex
				◦ Confusion			◦ Neuronal loss and astrocytosis of anterior horn cells
Nowacki *et al*., 2000 [8]	M	82	2	◦ Memory loss	3 weeks	4 weeks after symptomatic onset	◦ Extensive spongiform change and neuronal loss
				◦ Confusion			◦ Loss of anterior horn cells
Esteban *et al*., 2001 [4]	M	62	18	◦ Memory loss	4 months	10 months after symptomatic onset	◦ Neocortical and hipppocampal spongiosis
							◦ Severe neuronal loss with gliosis in the amygdala
							◦ Loss of anterior horn cells
Kovacs *et al*., 2002 [7]	M	62	8	◦ Numbness in left foot	5 months	7 months after symptomatic onset	◦ Extensive spongiform change, neuronal loss and astrocytosis
							◦ Reduced neuronal density in ventral horn
Niewiadomska *et al*., 2002 [5]	M	67	10.5	ND	ND	ND	ND
Niewiadomska *et al*., 2002 [5]	M	65	24	ND	ND	ND	ND
Niewiadomska *et al*., 2002 [5]	M	60	9	ND	ND	ND	ND
Panegyres *et al*., 2013	M	70	1.2	◦ Limb weakness and falls	At onset	First sign at clinical presentation	◦ Extensive spongiform change, neuronal loss and astrocytosis in the neocortex, with 12F10 synaptic immunopositivity in cerebral cortex, thalamus, cerebellar cortex and brainstem motor neurons.

*ND* not described.

Amyotrophy appeared in the terminal stages of CJD for the majority of these patients and was not an early or sole presenting feature as in our patient (Table 2). For these patients, prion protein propagation appeared to descend the corticospinal tract with rapidity from the brain, to the cervical spine and then trunk musculature; that is, in contrast to the ascending pattern of neurological dysfunction witnessed in our patient. For one patient, however, amyotrophic features were evident at initial presentation and remained prominent throughout the course of his disease [9]; marked cognitive deficits including memory loss, confabulation and constructional apraxia developed concurrently – distinct from our patient who developed dementia 10 days after the onset of amyotrophy.

Anterior horn cells and spinal ganglia are rarely investigated, but may explain amyotrophy and peripheral denervation in some patients with sporadic CJD [5]. Significant anterior horn cell loss was verified in five patients [4,8-10], in conjunction with diagnostic spongiform degeneration in the pyramidal tracts. One patient presenting with neurosensory deficits demonstrated decreased motor neuron density in the ventral horns [7]. Due to the restrictions placed upon our mortuary techniques, our patient’s spinal cord could not be microscopically evaluated. Nevertheless, clinical evidence of ascending anterior horn cell involvement was suggestive of upward prion propagation.

Supranuclear palsy of upgaze is sometimes found in other neurodegenerative diseases like progressive supranuclear palsy, but is rarely a feature of sporadic CJD [11]. Moreover, our patient’s development of catalepsy in the later stages of the disease is further evidence of ascending neurodegeneration.

The absence of upper motor neuron signs, the lack of bulbar involvement and the rapid progression negate the possible coexistence of amyotrophic lateral sclerosis (ALS) and CJD. Furthermore, the findings in our patient do not fulfill recognized diagnostic criteria for definite, probable or possible ALS using the El Escorial, Airlie House or Awaji-shima guidelines [12,13].

Huntington’s disease has been reported as presenting with motor neuron disease, suggesting that anterior horn cells are vulnerable to the effects of neurodegenerative processes of other etiologies including trinucleotide repeat disorder with protein misfolding, impaired deoxyribonucleic acid (DNA) transcription, ribonucleic acid (RNA) processing interference, apoptosis, cytoplasmic element dysfunction and glutamate excitotoxicity [14].

## Conclusion

This report provides a narrative of our patient’s experience of CJD from onset to death in just 36 days. In contrast to previously reported cases in the literature, amyotrophic features were the first clinical signs of the disease in the absence of dementia. Interestingly, prion propagation seems to have originated in the anterior horn cells, ascended the corticospinal tracts and eventually spread to the brain.

## Consent

Written informed consent was obtained from the patient’s next-of-kin for publication of this case report and any accompanying images. A copy of the written consent is available for review by the Editor-in-Chief of this journal.

## Competing interests

The authors have no competing interests to declare.

## Authors’ contributions

EA, RS and PKP analyzed and interpreted the patient data. PKP and RS diagnosed and managed the patient. All authors contributed to writing the paper and approved the final manuscript.
